# Motivational trade-offs and modulation of nociception in bumblebees

**DOI:** 10.1073/pnas.2205821119

**Published:** 2022-07-26

**Authors:** Matilda Gibbons, Elisabetta Versace, Andrew Crump, Bartosz Baran, Lars Chittka

**Affiliations:** ^a^School of Biological and Behavioural Sciences, Queen Mary University of London, London E1 4NS, United Kingdom;; ^b^Centre for Philosophy of Natural and Social Science, London School of Economics and Political Science, London WC2A 2AE, United Kingdom;; ^c^Faculty of Natural Science, University of Silesia, Katowice 40-007, Poland

**Keywords:** pain, nociception, insects, ethics, adaptation

## Abstract

Insects are traditionally thought to respond to noxious stimuli in an inflexible manner, without the ability to modulate their behavior according to context. We investigated whether bumblebees’ attraction to high sucrose solution concentrations reduces their avoidance of noxious heat. Bees were given the choice between either unheated or noxiously heated (55 °C) feeders with different sucrose concentrations and marked by different colors. Bees avoided noxious feeders when the unheated feeders contained high sucrose concentrations, but progressively increased feeding from noxious feeders when the sucrose concentration at unheated feeders decreased. This shows a motivational trade-off of nociceptive responses. Bees used learned color cues for their decisions, and thus the trade-off was based on processing in the brain, rather than just peripheral processing. Therefore, bees can use contextual information to modulate nociceptive behavior. This ability is consistent with a capacity for pain experiences in insects.

Nociception and nocifensive behavior—the detection of and response to noxious stimuli—occur across many animal taxa, including insects (e.g., ref. 1). In mammals, neurons descending from the brain can modulate nociception and nocifensive behavior (2). The adaptive function of reducing nociception is to ensure that the subjective feeling of pain does not compromise the animal’s performance in acquiring another motivational requirement (2, 3). For example, if an animal is food-deprived and sustaining injuries from fighting with its prey, reducing nociception and pain could improve fighting performance, and thus the chance of alleviating starvation. In mammals, mental states can also drive reduction of nocifensive behavior. For example, placebo effects, or imagining analgesia, reduces nociception and pain in humans (4). We test whether an insect, the bumblebee (*Bombus terrestris*), is capable of context-dependent, centrally controlled reduction of nocifensive behavior.

We used a motivational trade-off paradigm, where animals must flexibly trade-off two competing motivations. For example, hermit crabs require higher voltages of electric shock to evacuate their preferred shell species than for a less preferred one (5, 6). Shock avoidance is traded off against shell preference. Bees also display motivational trade-offs in non-noxious contexts (e.g., ref. 7). However, whether bees trade off noxious stimuli with other priorities is unknown.

We expanded on the motivational trade-off paradigm by ensuring that the trade-off relied on conditioned cues associated with the motivational stimuli, rather than direct sensory experience of the stimuli themselves. Bees were given the choice between two high-quality feeders (containing 40% sucrose solution) and two alternative feeders. In the latter, different groups of bees were offered 10%, 20%, 30%, or 40% sucrose solution (Fig. 1). Each group experienced the unheated temperature condition, with all feeders at room temperature (unheated), followed by the heated temperature condition, with the high-quality feeders at 55 °C (heated) and the alternative feeders unheated. We predicted that, when the noxiously heated feeders dispensed higher sucrose concentrations than the unheated feeders, bees would have a lower tendency to avoid noxiously heated feeders.

**Fig. 1.**
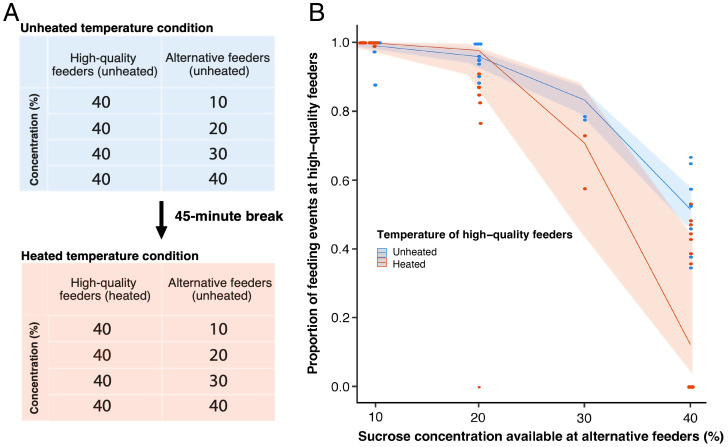
Experimental protocol and demonstration that bumblebees can prioritize high-quality food over noxious stimuli, depending on available alternative options. (*A*) Order in which the temperature conditions were experienced; temperature of the feeders in each temperature condition; sucrose concentration of feeders. Each of four different groups of bees (corresponding to one horizontal line in the tables) experienced only one combination of concentrations in each phase. Color and positions of feeders were counterbalanced. (*B*) Proportion of feeding events on high-quality feeders in both temperature conditions and all concentration conditions (*n* = 32). Lines represent the predictions of the generalized linear model. Shaded areas represent 95% confidence intervals.

## Results

Bees preferred unheated high-quality feeders (40% sucrose solution) when alternative feeders contained lower sucrose concentrations (10%, 20%, or 30%; *z* = −13.12, *P* < 0.001, *n* = 41) (blue data in Fig. 1*B*). However, when both feeders were high-quality and one of them was heated, bees tended to avoid the heated feeder (mean proportion of feeding events on heatable feeder: 0.5 unheated, 0.3 heated; *z* = −4.050, *P* < 0.001, *n* = 10) (pink data in Fig. 1*B*). In both the unheated and heated conditions, the proportion of feeding events on 40% feeders decreased as the sucrose concentration of alternative feeders increased (Fig. 1*B*). However, this decrease was greater when the 40% feeders were heated (*z* = −2.068, *P* = 0.039, *n* = 32) (compare pink data vs. blue data in Fig. 1*B*). Thus, bees traded off their motivation to avoid noxious heat against their preference for high sucrose concentrations.

We also recorded the number of “landing but not feeding” episodes on all feeders in the first and final foraging bouts. Unscented sucrose solution concentration cannot be detected unless the bee tasted the solution (8). The mean number of landing but not feeding episodes on all feeders was significantly lower in the final foraging bout compared to the first (mean number of landing events: 2.45 in first bout, 1.09 in final bout; *z* = −5.62, *P* < 0.001, *n* = 32). This reduction in landing but not feeding events between the first and final bout indicates that bees learned to use color/spatial cues, rather than landing on every feeder and sensing the sucrose/heat directly.

## Discussion

Bumblebees avoided noxiously heated feeders less when these dispensed higher sucrose concentrations than unheated feeders. Unlike trade-offs described in other invertebrates (5, 6, 9), this trade-off relied on associative memories, rather than direct experience of the stimuli. Bees’ ability to trade-off heat avoidance against sucrose preference indicates that conditioned motivational stimuli can influence nocifensive behavior, and the trade-off is mediated in the central nervous system (10, 11). As in other animals, such an ability is viewed as consistent with the capacity to feel pain (12–14), although because of the subjective nature of pain experience, it is not a formal proof. Nonetheless, given the potential ethical implications of our research, the precautionary principle dictates that the possibility of insect pain and suffering should be taken seriously in insect research laboratories as well as insect conservation (14, 15).

## Materials and Methods

### Experimental Set-Up.

**Fig. 2.**
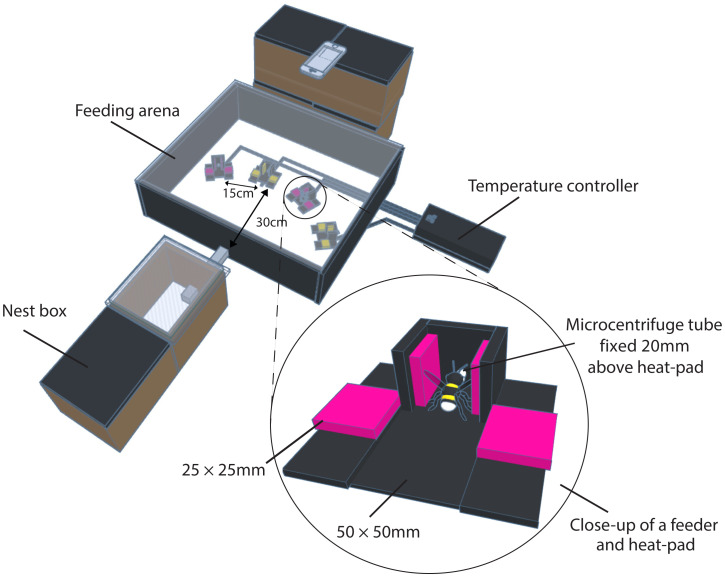
Testing arena set-up (not to scale). Four feeders on the floor of the arena, 30 cm from the entrance, 15-cm apart. The arena could be accessed from nest box via a Perspex corridor. A temperature controller was outside the arena and controlled the temperature of each feeder. The iPhone was placed above. Black circle shows one feeder close up, with the black heat-pad (50 cm × 50 cm), colored Perspex squares (25 cm × 25 cm), and a 1.5-mL microcentrifuge tube (with the end cut off) 20 mm above the heat-pad. Bee is shown in a feeding position.

Forty-one forager bees from eight colonies were tested. Bumblebee nests (Biobest, Belgium) were kept in boxes connected to the testing arena, which contained four feeders (Fig. 2). Each feeder was on top of a heat-pad, and had four Perspex squares (25 mm × 25 mm) colored either pink to the human eye (bee blue; hereafter “pink”) and human yellow (bee green; hereafter “yellow”) on a black background. Feeders were arranged in a semicircle, 15 cm apart and 30 cm from the arena entrance, with alternating colors. One color marked the two high-quality feeders, and the other color marked the two alternative feeders; colors were counterbalanced between bees.

### Training and Testing.

The bees first underwent training to familiarize them with the feeder contents (*SI Appendix*). Before testing, a forager bee was chosen and only that bee was let into the arena. The bee was allowed to feed from the four feeders, which each contained 20 μL of sucrose solution. Sucrose solution concentrations were calculated weight per weight (wt/wt).

First, all feeders were unheated (unheated temperature condition). Feeders were refilled every time the bee emptied them and landed on another feeder. Each bee completed 5 to 10 bouts (nest–arena–nest cycle) until it stopped foraging (no attempt to re-enter the arena for over 20 min). After a 45-min break to allow heat-pads to heat up, the bees entered the second testing stage (the heated temperature condition), where two of the high-quality feeders were heated to 55 °C (temperature was recorded using an infrared camera and infrared thermometer). The same testing protocol was followed.

Arena and feeders were cleaned with 70% ethanol every three bouts, as well as during the 45-min break. We recorded the number of feeding events, defined as “proboscis extended and contacting the sucrose-dispensing part of the feeder for more than 3 s,” and landing events, defined as “enters cardboard chamber of the feeder and does not feed,” using an iPhone 6s (Apple). For each bee, we calculated the mean proportion (number of feeding events on the high-quality feeders relative to the number of feeding events on all feeders) across all her foraging bouts for each temperature condition. We also calculated the total number of landing events per first and final foraging bouts for each bee. We analyzed the data in R (16), using generalized linear mixed-effect models (*SI Appendix*). All study materials, protocols, and methods are included in the main text and *SI Appendix*.

## Supplementary Material

Supplementary File

## Data Availability

Code and data have been deposited in Figshare: https://figshare.com/collections/Motivational_trade-off_code_and_data/6066371/2, (17).

## References

[1] L. Zhong, R. Y. Hwang, W. D. Tracey, Pickpocket is a DEG/ENaC protein required for mechanical nociception in *Drosophila* *larvae*. Curr. Biol. 20, 429–434 (2010). 20171104 10.1016/j.cub.2009.12.057 PMC2995491

[2] M. J. Millan, Descending control of pain. Prog. Neurobiol. 66, 355–474 (2002). 12034378 10.1016/s0301-0082(02)00009-6

[3] H. Ohashi, T. Sakai, Leucokinin signaling regulates hunger-driven reduction of behavioral responses to noxious heat in *Drosophila*. Biochem. Biophys. Res. Commun. 499, 221–226 (2018). 29559237 10.1016/j.bbrc.2018.03.132

[4] K. Hayashi, S. Aono, Y. Shiro, T. Ushida, Effects of virtual reality-based exercise imagery on pain in healthy individuals. BioMed Res. Int. 2019, 5021914 (2019). 31119173 10.1155/2019/5021914 PMC6500693

[5] M. Appel, R. W. Elwood, Motivational trade-offs and potential pain experience in hermit crabs. Appl. Anim. Behav. Sci. 119, 120–124 (2009).

[6] R. W. Elwood, M. Appel, Pain experience in hermit crabs? Anim. Behav. 77, 1243–1246 (2009).

[7] P. Schmid-Hempel, R. Schmid-Hempel, Efficient nectar-collecting by honeybees II. Response to factors determining nectar availability. J. Anim. Ecol. 56, 219–227 (1987).

[8] H. M. Whitney, A. Dyer, L. Chittka, S. A. Rands, B. J. Glover, The interaction of temperature and sucrose concentration on foraging preferences in bumblebees. Naturwissenschaften 95, 845–850 (2008). 18523748 10.1007/s00114-008-0393-9

[9] Y. Shinkai , Behavioral choice between conflicting alternatives is regulated by a receptor guanylyl cyclase, GCY-28, and a receptor tyrosine kinase, SCD-2, in AIA interneurons of *Caenorhabditis elegans*. J. Neurosci. 31, 3007–3015 (2011). 21414922 10.1523/JNEUROSCI.4691-10.2011 PMC6623760

[10] K. Vogt , Shared mushroom body circuits underlie visual and olfactory memories in *Drosophila*. eLife 3, e02395 (2014). 25139953 10.7554/eLife.02395 PMC4135349

[11] N. Mancini, M. Giurfa, J.-C. Sandoz, A. Avarguès-Weber, Aminergic neuromodulation of associative visual learning in harnessed honey bees. Neurobiol. Learn. Mem. 155, 556–567 (2018). 29793042 10.1016/j.nlm.2018.05.014

[12] E. Walters, Defining pain and painful sentience in animals. Anim. Sentience 21, 10.51291/2377-7478.1360 (2018).

[13] R. W. Elwood, Discrimination between nociceptive reflexes and more complex responses consistent with pain in crustaceans. Philos. Trans. R. Soc. Lond. B Biol. Sci. 374, 20190368 (2019). 31544604 10.1098/rstb.2019.0368 PMC6790375

[14] L. U. Sneddon, R. W. Elwood, S. A. Adamo, M. C. Leach, Defining and assessing animal pain. Anim. Behav. 97, 201–212 (2014).

[15] J. Birch, Animal sentience and the precautionary principle. Anim. Sentience 16, 10.51291/2377-7478.1200 (2017).

[16] R Core Team, R: A Language and Environment for Statistical Computing (R Foundation for Statistical Computing, Vienna, Austria, 2021).

[17] M. Gibbons, Motivational trade-offs and modulation of nociception in bumblebees. figshare. https://figshare.com/collections/Motivational_trade-off_code_and_data/6066371/2. Deposited 27 June 2022. 10.1073/pnas.2205821119 PMC9351458 35881793

