# Supplementary material for: Motivational trade-offs and modulation of nociception in bumblebees
Source: Proc Natl Acad Sci U S A. 2022 Jul 26;119(31):e2205821119. doi: 10.1073/pnas.2205821119 (PMC9351458; doi:10.1073/pnas.2205821119)
Supplement: Supplementary File [file pnas.2205821119.sapp.pdf]

Supporting Information

Animal care

Insects are not protected under animal welfare law in the United Kingdom, but we followed the Association for the Study of Animal Behaviour's Guidelines for the Use of Animals in Research (1). Relatively few bees were tested (N = 41). Moreover, although the experiment involved noxiously-heated feeders, bees were never forced onto these – food was always available at unheated feeders.

Bees were kept in either 40×28×11 cm or 28×16×11 cm two-part wooden boxes connected via a Perspex tunnel (25 cm length; 3.5 × 3.5cm cross-section) to the testing arena (Perspex-roofed flight arena; 56cm × 56cm), which had four feeders (see Figure 2). One half of the two-part boxes that colonies were kept in contained the nest and had a wooden top to maintain 24-hour darkness. The other half had a 1cm-deep gravel substrate and a Perspex top, with a 12:12 hour light:dark cycle. A 1 cm-diameter hole connected the two sections. For ventilation, boxes also had four 2 cm-diameter exterior holes, which were covered with gauze to prevent escapes. Temperature was maintained at 23 °C. Colonies received seven grams of pollen (Natupol Pollen, Koppert Biological Systems, UK) every two days.

Heat-pads

Heat-pads were custom-built using a single side copper-clad (copper thickness 70μm) standard printed circuit board laminate (made of woven fiber-glass and epoxy resin, 1.57mm thick, 50mm × 50mm each) with an etched resistive heating element consisting of 18 loops (path width 0.6mm). The surface of each heat-pad was fitted with LM35 temperature sensors with thermally conductive adhesive (AG TermoGlue, TermoPasty, Poland). Heat-pads were powered with 12V DC, regulated by TIP122 transistors, driven with Arduino Uno Rev3. The temperature readouts were smoothed with a Kalman filter (SimpleKalmanFilter library).

Training and testing

We identified foragers as bees that left the nest to feed more than four times in ~30 minutes, and marked these on the dorsal thorax with either an Opalith number tag (using Loctite Super Glue) or a colored Uni POSCA marker pen. Before testing, we

removed the bees from the arena using a plastic cup and a Perspex square to catch each bee and return her to the nest. We then cleaned the arena and feeders with 70% ethanol solution. When tests were not being run, bees underwent group training, in which the whole colony could access cotton wool soaked with sucrose solution on the feeders in the arena. Then, after a forager was chosen, they underwent an individual training phase that consisted of the feeders not being refilled until the bee had consumed the sucrose solution from all four feeders, to ensure that she had experience of every feeder. During testing, if a forager spent five minutes in the testing arena without feeding, we returned her to the nest. Heat pad temperature was recorded using an infrared camera (FLIR One Infrared Camera, USA) and infrared thermometer (CASON CA380 Infrared Thermometer, TMS Europe). Feeder color and order were counterbalanced (high-quality feeders were pink for 20 subjects, and yellow for 21 subjects; the position of the feeders from left to right was 'pink, yellow, pink, yellow' for 18 subjects and the opposite for 23 subjects).

Inclusion criteria

Our hypothesis relied on the bees distinguishing between the two concentrations of sucrose solutions and reliably choosing the higher one. The inclusion criterion for concentration conditions 10%, 20% or 30% was that the proportion of feeding events at the feeders containing 40% had to be significantly different from 0.5. The inclusion criterion for concentration condition 40% was that the proportion of feeding events at the feeders containing 40% had to not be significantly different from 0.5. One bee from the 20% condition and eight bees from the 30% condition did not meet our inclusion criterion and were excluded, giving a total sample size of 32 bees.

Statistical analysis

We analyzed the data in R (R Core Team, Cran-r-project, Vienna, Austria, version 1.3.1093), using generalized linear mixed effect models (GLMMs; packages: "lme4", (2) "car" (3). We identified the most parsimonious models through the ANOVA function and stepwise backward elimination. As our data were proportions, and thus binary, we used a binomial distribution and logit link function. We checked model assumptions using histograms and Q-Q plots, and considered $p < 0.05$ significant.

Four separate GLMMs were fitted. In the first model, we tested whether bees preferred unheated feeders to heated feeders. The response variable was proportion of feeds on the heatable feeder; the fixed effect was temperature condition (heated, unheated); and the random effects were bee ID nested in colony ID. Model simplification removed location and color of the heatable feeder and bout number. In the second model, we tested whether bees preferred 40% sucrose concentration feeders to lower-concentration alternative feeders. The response variable was the proportion of feeding events on the heatable feeder, and the fixed effect was concentration condition (10%, 20%, 30%, 40%). Model simplification removed location and color of the heatable feeder, bout number, and colony ID. In the third model, we tested whether bees trade-off their heat aversion against their preference for higher sucrose concentrations. The response variable was proportion of feeding events on the heatable feeder; the fixed effects were temperature condition, concentration condition, and the temperature × concentration interaction; and the random effect was bee ID for the temperature condition only (because only the temperature condition was repeated measures). Model simplification removed location and color of the heatable feeder, bout number, and colony ID. In the fourth model, we tested whether landing events increased significantly in the final bout from the first bout. The response variable was the number of landing events per bee; the fixed effect was bout; the random effect was bee ID.

Supporting information references

1. W.M.S. Russell and R.L., Burch, The principles of humane experimental technique. *Methuen*. (1959).
2. D. Bates, M. Mächler, B. Bolker and S. Walker, Fitting linear mixed-effects models using lme4. *J. Stat. Softw.* 67, 1–48. (2015).
3. J. Fox, S. Weisberg, B. Price, D. Adler, Bates, G. Baud-Bovy, B. Bolker, S. Ellison, D. Firth, M. Friendly, and G.Gorjanc, Package ‘car’. (2021).
